# Supplementary material for: Quantifying Human Mobility Perturbation and Resilience in Hurricane Sandy
Source: PLoS One. 2014 Nov 19;9(11):e112608. doi: 10.1371/journal.pone.0112608 (PMC4237337; doi:10.1371/journal.pone.0112608)
Supplement: Table S1 — Data Volume for Each 24-Hour Period. (DOC) [file pone.0112608.s001.doc]

**Supporting Information Table S1**

**Table S1.** Data Volume for Each 24-Hour Period

| Time Period | Number of Tweets |
| --- | --- |
| Day 1 | 63,086 |
| Day 2 | 49,373 |
| Day 3 | 55,864 |
| Day 4 | 52,448 |
| Day 5 | 65,800 |
| Day 6 | 60,230 |
| Day 7 | 54,403 |
| Day 8 | 57,575 |
| Day 9 | 57,813 |
| Day 10 | 58,769 |
| Day 11 | 50,543 |
| Day 12 | 55,730 |
