# Supplementary material for: Quantifying Human Mobility Perturbation and Resilience in Hurricane Sandy
Source: PLoS One. 2014 Nov 19;9(11):e112608. doi: 10.1371/journal.pone.0112608 (PMC4237337; doi:10.1371/journal.pone.0112608)
Supplement: Table S2 — Displacement Distribution. (DOC) [file pone.0112608.s002.doc]

**Supporting Information Table S2**

**Table S2.** Displacement Distribution

| Days | 1-100m | 100-500m | 500m-1km | 1-5km | 5-10km | >10km |
| --- | --- | --- | --- | --- | --- | --- |
| 1 | 23494 | 4949 | 1900 | 1713 | 254 | 294 |
| 2 | 16080 | 3040 | 1320 | 1920 | 453 | 367 |
| 3 | 18136 | 4057 | 1722 | 2602 | 563 | 403 |
| 4 | 17661 | 3593 | 1530 | 2230 | 559 | 382 |
| 5 | 21547 | 4407 | 1960 | 3004 | 674 | 424 |
| 6 | 20819 | 4051 | 1846 | 2671 | 561 | 409 |
| 7 | 18532 | 3725 | 1728 | 2313 | 597 | 501 |
| 8 | 18243 | 3711 | 1779 | 2731 | 886 | 745 |
| 9 | 18769 | 3681 | 1710 | 2730 | 824 | 639 |
| 10 | 20144 | 3689 | 1729 | 2458 | 612 | 538 |
| 11 | 15961 | 3302 | 1485 | 2310 | 702 | 571 |
| 12 | 15875 | 3782 | 1827 | 2948 | 917 | 795 |
