# Supplementary material for: Quantifying Human Mobility Perturbation and Resilience in Hurricane Sandy
Source: PLoS One. 2014 Nov 19;9(11):e112608. doi: 10.1371/journal.pone.0112608 (PMC4237337; doi:10.1371/journal.pone.0112608)
Supplement: Table S3 — Displacement Fitting Results. (DOC) [file pone.0112608.s003.doc]

**Supporting Information Table S3**

**Table S3.** Displacement Fitting Results

| Period | *β* Value | *λ* Value | *κ* Value (m) |  | *KS*-test | Lognormal Comparison | Exponential Comparison |
| --- | --- | --- | --- | --- | --- | --- | --- |
| Day 1 | 1.73 | 2.70E-05 | 591 |  | 0.025^***^ | 26.58^***^ | 1251.28^***^ |
| Day 2 | 1.24 | 5.56E-05 | 4 |  | 0.023^***^ | 556.90^***^ | 43925.59^***^ |
| Day 3 | 1.20 | 5.23E-05 | 4 |  | 0.022^***^ | 598.50^***^ | 36351.78^***^ |
| Day 4 | 1.13 | 6.01E-05 | 3 |  | 0.015^**^ | 816.76^***^ | 39808.21^***^ |
| Day 5 | 1.25 | 5.78E-05 | 4 |  | 0.025^***^ | 446.96^***^ | 34612.09^***^ |
| Day 6 | 1.20 | 7.79E-05 | 4 |  | 0.022^**^ | 621.02^***^ | 36960.03^***^ |
| Day 7 | 1.23 | 6.44E-05 | 4 |  | 0.022^*^ | 492.43^***^ | 37638.76^***^ |
| Day 8 | 1.21 | 7.40E-05 | 4 |  | 0.021^*^ | 663.75^***^ | 44210.40^***^ |
| Day 9 | 1.25 | 6.63E-05 | 5 |  | 0.030^*^ | 621.45^***^ | 39790.81^***^ |
| Day 10 | 1.22 | 6.10E-05 | 4 |  | 0.022^***^ | 528.08^***^ | 40092.48^***^ |
| Day 11 | 1.20 | 4.98E-05 | 4 |  | 0.030^***^ | 822.63^***^ | 42010.51^***^ |
| Day 12 | 1.21 | 5.31E-05 | 4 |  | 0.021^***^ | 722.15^***^ | 42531.20^***^ |

** p*-value<0.05, ** *p*-value<0.01, *** *p*-value<0.001
