# Supplementary material for: Quantifying Human Mobility Perturbation and Resilience in Hurricane Sandy
Source: PLoS One. 2014 Nov 19;9(11):e112608. doi: 10.1371/journal.pone.0112608 (PMC4237337; doi:10.1371/journal.pone.0112608)
Supplement: Table S4 — Fitting Results between the Center of Mass and the Radius of Gyration. (DOC) [file pone.0112608.s004.doc]

**Supporting Information Table S4**

**Table S4.** Fitting Results between the Center of Mass and the Radius of Gyration

| Relation | Coefficient Estimation | | Std. Error | *t*-value |
| --- | --- | --- | --- | --- |
| *Δd_CM_* | Intercept Value | 0.611 | 0.0595 | 10.28^***^ |
|  | $r_{g}^{N}$coefficient | 0.456 | 0.0276 | 16.55^***^ |
| $r_{g}^{P}$ | Intercept Value | 1.345 | 0.296 | 4.54^***^ |
|  | $r_{g}^{N}$coefficient | 0.276 | 0.087 | 3.16^***^ |

** p*-value<0.05, ** *p*-value <0.01, *** *p*-value <0.001
