# Supplementary material for: Quantifying Human Mobility Perturbation and Resilience in Hurricane Sandy
Source: PLoS One. 2014 Nov 19;9(11):e112608. doi: 10.1371/journal.pone.0112608 (PMC4237337; doi:10.1371/journal.pone.0112608)
Supplement: Materials S1 — Detailed description of methods used for data collection and analysis. This also includes supporting findings and figures relating to the analytical results of the shifting distance of the center of movements and radius of gyrations. (DOC) [file pone.0112608.s005.doc]

**Materials S1**

**Supporting Information Methods**

Data Collection

Human mobility data were collected from Twitter. We used the open API from Twitter to create a continuous connection between a computer in our research lab and a streaming endpoint at a Twitter server. The connection continuously downloaded tweets in real-time. Each public tweet was collected if the tweet had geolocation information, also called being geotagged, and if the coordinate was within 74˚15’W to 73˚40’W longitude and 40˚30’N to 40˚57’N latitude. Thus, the data collection was ensured coverage of the entirety of New York City. Every tweet included the text information, the tweet’s ID, the name and ID of the user who posted the tweet, the time stamp when it was posted, and the coordinate. A reconnecting mechanism was coded so that if the streaming was lost for 30 seconds, a restart message was displayed and a new streaming connection established. We observed no reconnections during the 12 days of data collection. Despite power outages, we observed no break in our data. In fact, the number of tweets recorded during the first 24-hours after Hurricane Sandy struck the city was the second largest daily number of tweets collected over the 12 day data collection period, at 12 percent more than the average number of geotagged tweets sent during the ensuing 11 days. The amount of data collected each 24-hour period can be found in Table S1.

We analyzed the time elapsed between every two consecutive tweets from each individual, and found the time followed a truncated power-law distribution with the scale-invariance *β* value only slightly larger than 1, which is in line with the *β*=0.9±0.1 reported in a previous study [1]. The exponential cutoff *λ* was 3.93×10^-6^, and the minimum fitting value *κ* was 10 seconds. This distribution is plotted in Fig. S1.

Displacements Distribution Analysis

We found all the displacements using the retrieved movement trajectories. The distance between two coordinates was calculated using the Haversine formula [2]:

$$d=2r\times\sin^{-1}\left(\sqrt{\sin^{2}\left(\frac{\varphi_{2}-\varphi_{1}}{2}\right)+\cos\varphi_{1}\cos\varphi_{2}\sin^{2}\left(\frac{\phi_{2}-\phi_{1}}{2}\right)}\right)$$

Where *r* is the earth radius, which approximately equals to 6,367,000 meters, *ϕ* is the latitude, and *φ* is the longitude. We counted the numbers of trips within different ranges of distances, and results are shown in Fig. 2A and Table S2.

Then we fitted the displacement data to truncated power-law distribution [1,3]:

$$P(\Delta r)\propto\Delta r^{-\beta}e^{-\lambda\Delta r}$$

Where *Δr* is the displacement, *β* is the scaling parameter, *λ* is the exponential cutoff value. We found the displacement data in each 24-hour period followed a truncated power-law distribution. The results are shown in Fig. 2B and Table S2. To test if the truncated power-law distribution was the best fit, we conducted two tests: the Kolmogorov-Smirnov (*KS*) test, and Maximum Likelihood Estimation (MLE) to compare truncated power-law distribution to both exponential distribution and lognormal distribution [4]. The results are shown in Table S3. For each 24-hour period, the data sample passed the *KS* test, and therefore, the truncated power-law distribution had a high-level of goodness of fit. Also, the comparison results confirmed that the truncated power-law distribution is a better fit compared to both lognormal and exponential distributions.

Estimating the Shifting Distance of Center of Mass

The first parameter we used to capture the perturbation in human movement trajectory was the shift of center of mass. We first calculated the center of mass of movement trajectories $\vec{r}_{CM}^{P}$ during Hurricane Sandy using the following equation [1]:

where *n(t)* was the number of locations an individual visited in the 24-hour period, $\vec{r}_{i}$was the coordinate. Then we calculated the center of mass for each 24-hour period from Oct. 30, 2012 to Nov. 10, 2012 using the equation listed above. Using the average center of mass in the normal state and the center of mass in perturbation state, the shifting distance *Δd_CM_* was calculated using the following equation:

Where $\vec{r}_{CM}^{P}$ is the center of mass of a movement trajectory in the perturbation state, and $\vec{r}_{CM}^{N}$ is the center of mass in the normal state. Using both the KS test and distribution comparison, we found that the distribution of *Δd_CM_* followed a stretched exponential distribution and the results are shown in Fig. S2.

$$P(\Delta d_{CM})=e^{-\Delta d_{CM}^{0.655}}$$

Estimating the Radius of Gyration

The second parameter to describe perturbation of movement trajectories is the radius of gyration. Similar to the center of mass, we calculated the radius of gyration in each 24-hour period using the following equation (*2*)

Where $\vec{r}_{i}$ was the coordinate, and $\vec{r}_{CM}$was the center of mass of a movement trajectory in the each 24-hour period.

The radius of gyration in the perturbation states was calculated using 2241 active users from the first 24-hour period, and the radius of gyration in the steady states was the average values from Nov. 3 to Nov. 9. Both *r_g_^P^* and *r_g_^N^* followed truncated power-law distributions. The results of the fitting of the distributions are shown in Fig. S3.

Estimating Predictability

To understand if the perturbation of movement trajectories can be predicted, we first plotted *Δd_CM_* and *r_g_^N^* with logarithmic axes (Fig. 3A). The two parameters showed a positive correlation. To quantify the relation, we conducted a *t*-test between *Δd_CM_* and *r_g_^N^* to find the correlation coefficient. We also conducted linear regression to develop the equation between the two parameters. Results are shown in Fig. 3A and Table S4. We followed a similar procedure to find the relation between *r_g_^N^* and *r_g_^N^*. The results are shown in Fig. 3B and Table S4.

**Supporting Information Figures**

**
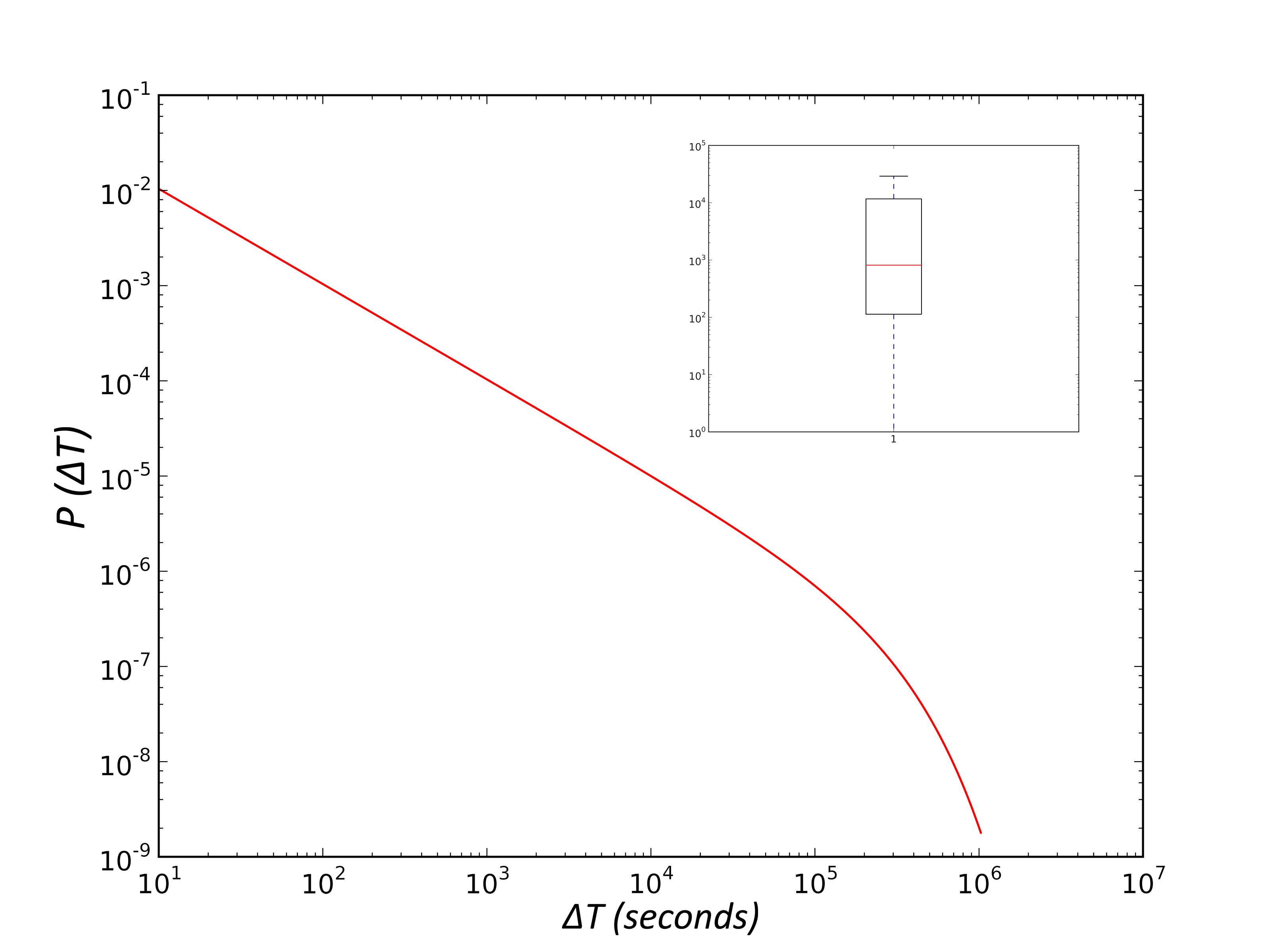
**

**Figure S1. Distribution of time intervals.** *ΔT* is the time interval between two consecutive tweets from the same individual. The distribution has *β*=1+6.3×10^-9^, *λ*=3.93×10^-6^, and *κ*=10 seconds. The inset is the box-and-whisker-plot of *ΔT*. The minimum value was 1 second, and the maximum value was 1,026,563 seconds (11.88 days). The median value was 813 seconds (13.55 minutes), the first quartile was 113 seconds (1.88 minutes), and third quartile was 11,677 seconds (3.24 hours). The whiskers show the 1.5 interquartile ranges.


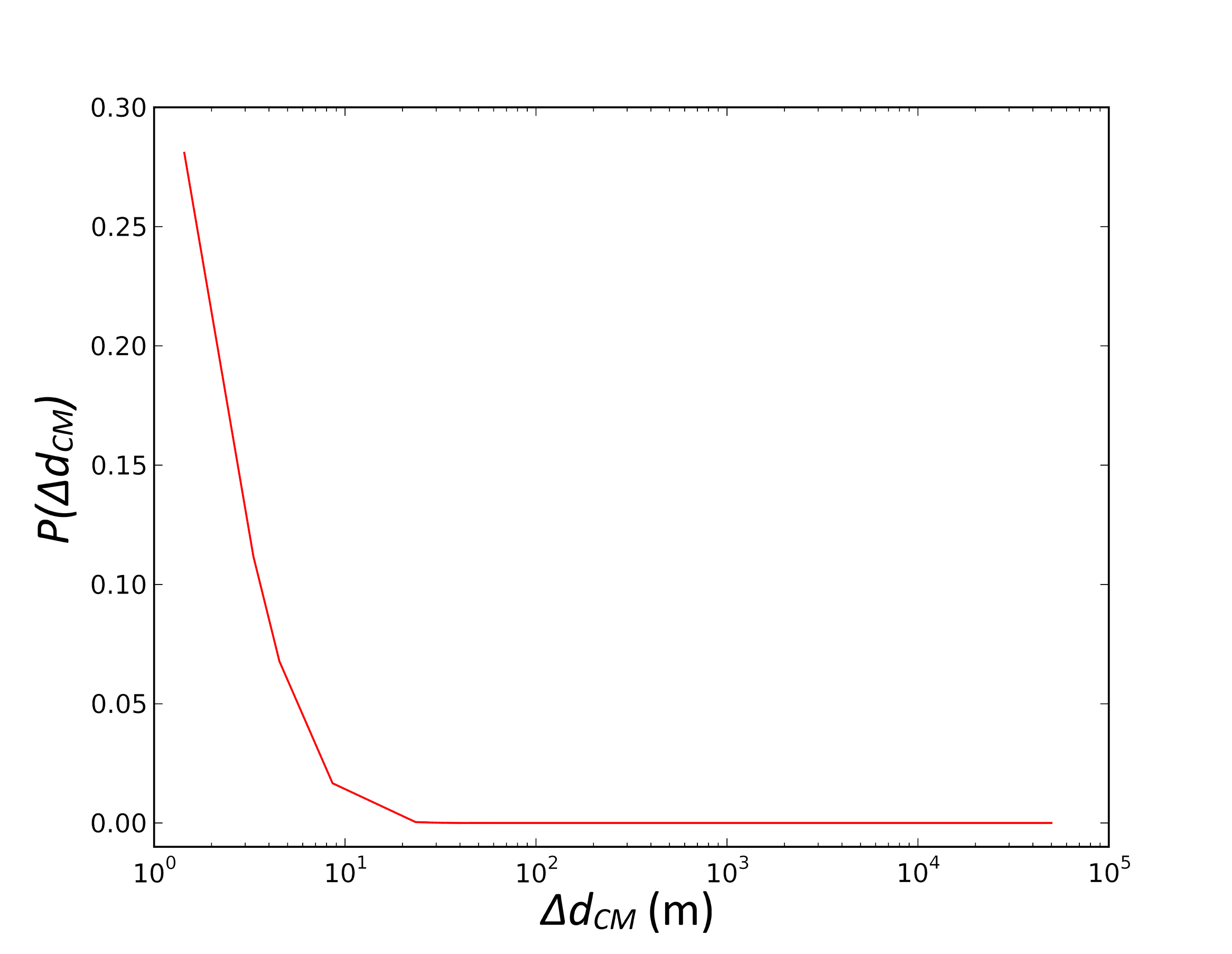


**Figure S2. Distribution of *Δd_CM_*.** The distribution followed a stretched exponential distribution. Distribution comparison between lognormal distribution and stretched exponential distribution returned *R*=-233.13 (p<0.001) which favors the latter. Also, the comparison between truncated power-law and stretched exponential distributions returned *R*=-1042.49 (*p*<0.001) which favors the latter as well.

**
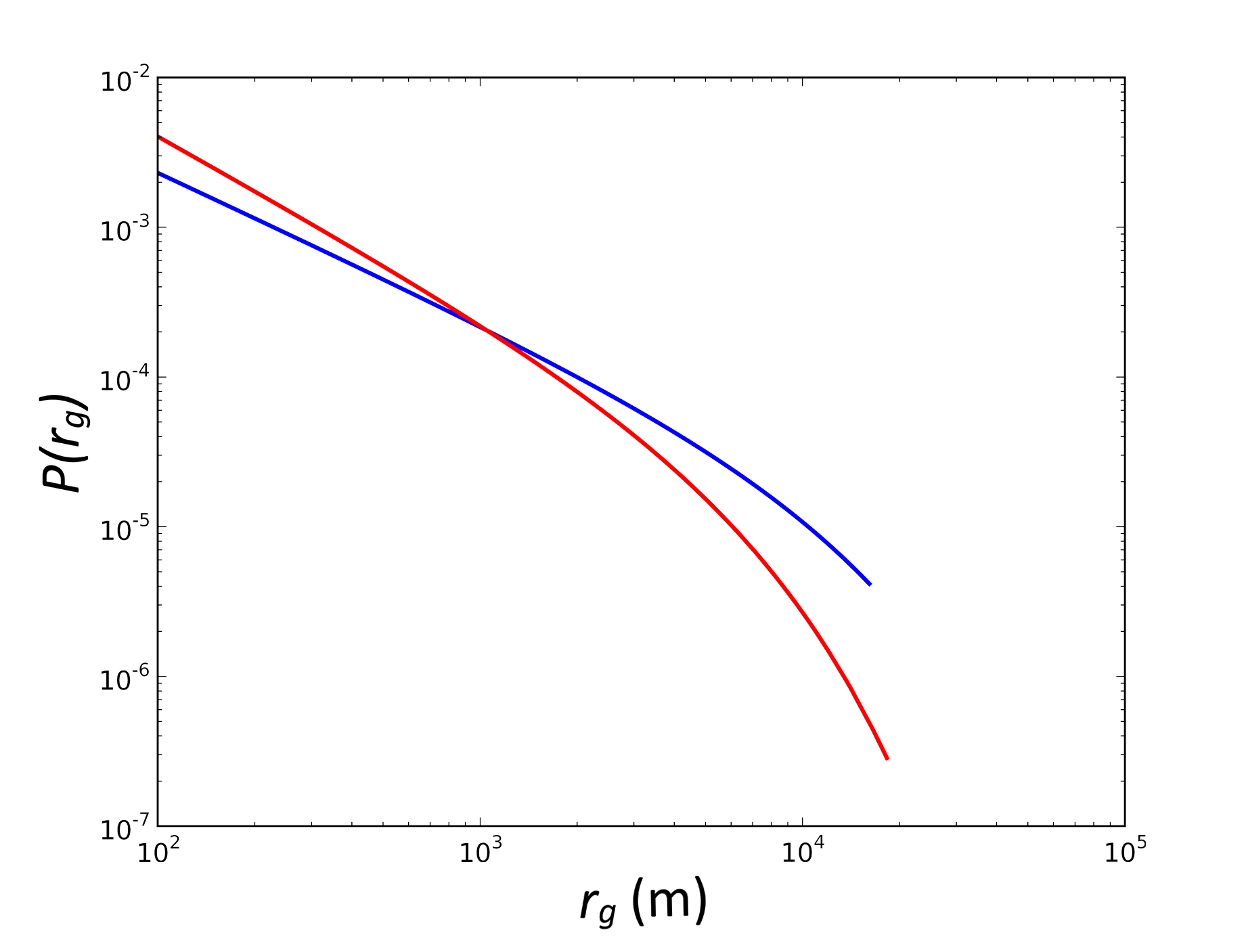
**

**Figure S3. Distribution of *r_g_*.** *r_g_^P^* followed truncated power-law distributions (red line). It has *β*=1.19 which is close to the *β* value (1.20) reported in [1], *λ*=1.84×10^-4^, and *κ*=100 m. However, *r_g_^N^* showed no such attribute (blue line).

**References**

1. González MC, Hidalgo CA, Barabási AL (2008) Understanding individual human mobility patterns. Nature 453: 779-782.
2. Robusto C (1957) The cosine-haversine formula. The American Mathematical Monthly 64: 38-40.
3. Clauset A, Shalizi CR, Newman ME (2009) Power-law distributions in empirical data. SIAM review 51: 661-703.
4. Klaus A, Yu S, Plenz D (2011) Statistical analyses support power law distributions found in neuronal avalanches. PLoS ONE. doi: 10.1371/journal.pone.0019779.
